# Eugène Delacroix (1798-1863). "Arab Horses Fighting in a Stable." 1860.

**DOI:** 10.3201/eid0808.020800

**Published:** 2002-08

**Authors:** Polyxeni Potter

**Affiliations:** * Centers for Disease Control and Prevention, Atlanta, Georgia, USA

**Figure:**
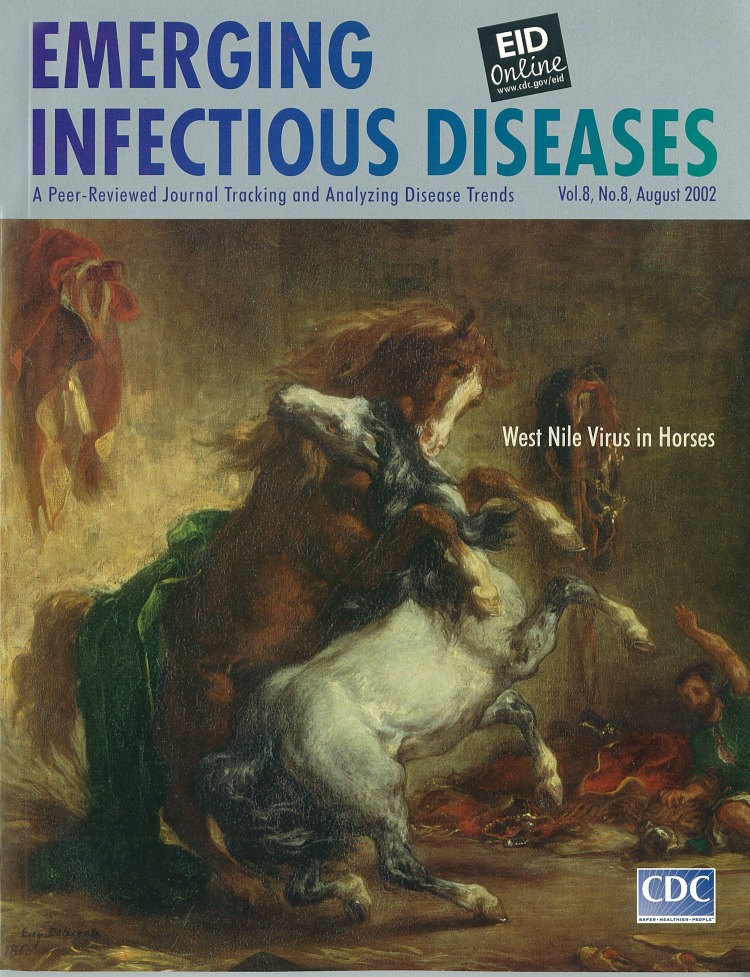
**Eugène Delacroix (1798-1863). "Arab Horses Fighting in a Stable." 1860. **Oil on canvas. Photo: Gerar Blot. Copyright Réunion des Musées Nationaux/Art Resource, NY Louvre, Paris, France

From his early years, Delacroix, like his contemporary Théodore Gericault, was attracted to the savagery of wild animals. In a note written in Morocco, Delacroix mentions a scene of fighting horses. Among the precedents for this kind of wild-animal imagery was the antique group "Lion Attacking a Horse" (Rome, Mus. Conserv.), which was said to have been particularly admired by Michelangelo and which was copied in stone by Peter Scheemakers (1740; Rousham Park, Oxon). George Stubbs used the wild-animal theme within naturalistic settings in several paintings, for example, in "Horse Attacked by a Lion," (1770; London, Tate), of which Gericault made at least one copy (1820/21; Paris, Louvre).

In "Arab Horses Fighting in a Stable," his version of the subject, Delacroix was able to synthesize the classical with the exotic, with his studies of ecorché (French for flayed bodies), with the example of English art, and with the work of Rubens—Delacroix owned Pieter Claesz Soutman's engravings of Rubens' paintings of hunts. In 1847, Delacroix described two of these engravings in detail, indicating how highly he valued the elements of movement, variety, and unity. Of Delacroix' three great lion hunts, three have survived, a fragment (1855, now in Bordeaux, France) and two complete paintings: one from 1858 (now in Boston, Massachusetts) and one from 1861 (now in Chicago, Illinois); the latter is the most spacious and free in its handling of circular, dancelike movements that suggest a perpetual struggle—one of the underlying themes in which form and content are inseparable.

From The Dictionary of Art, Macmillan, NY, NY, 1996.

